# Group A streptococcal SpeB modifies IgA through targeting regions other than the hinge

**DOI:** 10.1128/spectrum.02450-24

**Published:** 2025-03-25

**Authors:** Victoria Vassen, Emi Tanaka, Kirsten Moll, Christian Spoerry, Silvia Synowsky, Sally L. Shirran, Ulrich Schwarz-Linek, Edmund Loh, Mattias Svensson, Anna Norrby-Teglund

**Affiliations:** 1 Center for Infectious Medicine, Karolinska Institutet, Karolinska University Hospital Huddinge 167724, Huddinge, Stockholm County, Sweden; 2 Department of Microbiology, Tumor and Cell Biology, Karolinska Institute 27106 , Stockholm, Stockholm County, Sweden; 3 Biomedical Sciences Research Complex, University of St Andrews, North Haugh, St Andrews, Scotland, United Kingdom; Ludwig-Maximilians-Universitat München, Germany

**Keywords:** group A *Streptococcus*, IgA, protease, immune evasion, pathogenesis

## Abstract

Degradation of immunoglobulin (Ig) represents an important bacterial immune evasion strategy. For mucosal colonization, degradation of IgA is of particular importance, and many bacteria secrete specific IgA proteases that typically target the extended hinge region of IgA1. Such a specialized IgA protease has not yet been reported in Group A *Streptococcus* (GAS), despite its ability to successfully colonize human mucosal surfaces. In this study, we focused on the cysteine protease SpeB secreted by GAS and analyzed the interaction of SpeB with IgA. Assays using bacterial supernatants from wild-type and *speB*-deficient isogenic mutant strains, as well as recombinant SpeB, showed a SpeB-dependent IgA-modifying activity. SpeB resulted in the degradation of multimeric IgA, including the dimeric form, which was most notable in IgA2. The modification products were smaller in size than the heavy chain, suggesting a modification different from the classical hinge cleavage. Mass spectrometry analysis and glycosylation profiles indicated a putative cleavage in the C-terminal region, affecting the tailpiece and resulting in the loss of higher molecular weight multimeric/dimeric forms of IgA. Given the importance of dimeric IgA at mucosal surfaces, future studies are warranted to address whether IgA modification by SpeB represents a GAS immune evasion mechanism at this site.

**IMPORTANCE:**

Group A *Streptococcus* (GAS) is an important human pathogen with the ability to efficiently colonize mucosal surfaces and cause a wide spectrum of diseases ranging from pharyngotonsillitis to severe invasive infections or post-streptococcal sequelae. Immunoglobulins (Ig), in particular IgA, are critical effector molecules in the defense against pathogen colonization at mucosal surfaces. In this study, we focused on the cysteine protease SpeB, secreted by GAS, and investigated its interaction with human IgA. We report a SpeB-dependent IgA modification that involved the loss of multimeric/dimeric forms of IgA, predominantly affecting IgA2. The putative modification region is the C-terminus of IgA, which differs from the cleavage site of specialized IgA proteases targeting the hinge region. These findings suggest that IgA modification by SpeB might represent an immune evasion strategy utilized by GAS to colonize human mucosal tissue.

## INTRODUCTION

Group A *Streptococcus* (GAS) is a strictly human pathogen that can colonize the upper respiratory tract and skin, leading to either asymptomatic carriage or superficial, uncomplicated infections such as pharyngitis and impetigo (1, 2). However, it can also lead to severe, potentially life-threatening, invasive diseases such as bacteremia, streptococcal toxic shock syndrome, and necrotizing soft tissue infections (1, 2).

GAS has evolved several immune evasion mechanisms targeting the complement system, antimicrobial factors, cytokines, chemokines, and immunoglobulins (Ig) (2). Virulence factors targeting the latter include the well-characterized cysteine protease IdeS and the endoglycosidase EndoS, which exclusively modify IgG. IdeS cleaves IgG in the hinge region, impairing opsonophagocytosis (3), and the endoglycosidase EndoS hydrolyzes core glycans from the IgG heavy chain (HC), resulting in increased survival of GAS in the blood (4, 5).

IgA-specific proteases are common virulence factors among colonizers and pathogens of the respiratory tract. The proteases typically target the extended hinge region of IgA1 (6). IgA2 lacking this structure is therefore less susceptible to cleavage. The cleaved intact Fab portion can still bind to its antigen, but Fc-mediated effector functions, such as the loss of antibody-mediated agglutination and opsonophagocytosis, are inhibited (6–8). IgA1 proteases have been described for many colonizers of the oral cavity and pharynx, e.g., *Streptococcus sanguinis* (9, 10), *Streptococcus oralis* (11), and *Streptococcus mitis* (11), as well as pathogenic bacteria such as *Neisseria meningitidis* (12), *Haemophilus influenzae*, and *Streptococcus pneumoniae* (11, 13, 14). In GAS, it has been debated whether IgA cleavage is achieved through the cysteine protease SpeB with two contrasting reports (15, 16). While Collin et al. (15) reported that SpeB cleaved Ig of different isotypes, including IgA, Persson et al. (16) argued that this occurred only under reducing nonphysiologic conditions.

In this study, we have analyzed the interaction of SpeB with human IgA. We show that human serum IgA is modified by SpeB under physiological conditions. Furthermore, SpeB cleavage resulted in the loss of multimeric/dimeric forms of IgA and predominantly affected IgA2.

## MATERIALS AND METHODS

### Bacterial strains and growth conditions

GAS strain 5448, isolated from a patient with invasive infection (17), and its isogenic 5448Δ*spe*B mutant are generated by allelic exchange mutagenesis as previously detailed in (18). Both strains were provided by Dr. Malak Kotb. Bacterial strains were cultured in Todd Hewitt broth supplemented with 1.5% yeast extract (THY) at 37°C until the stationary phase.

### Ammonium sulfate precipitation

Twenty-five milliliters of bacterial overnight cultures were centrifuged, and supernatants were precipitated at 4°C. A 25 mL volume of 4M ammonium sulfate was added dropwise to the stirring supernatants and further stirred for 3.5 hours. The precipitated supernatants were centrifuged at 20,000 × *g* for 15 minutes at 4°C, and the pellets were resuspended in 1 mL PBS. The buffer was exchanged with PBS by Zeba Spin Micro Desalting Columns (Thermo Fisher Scientific), and the concentrated supernatants were stored at −80°C.

### Ig degradation assay using GAS supernatant

Concentrated bacterial supernatants from stationary-phase bacteria cultured in THY were prepared by ammonium sulfate precipitation, as described above. A 10 µg sample of human serum IgA (Sigma-Aldrich) was incubated with a 1:5 diluted concentrated supernatant in PBS for 16 hours at 37°C, and samples were stored at −20°C. Protease inhibitors (Protease Inhibitors Set 786-207, G-Biosciences) were individually incubated for 15 minutes with concentrated supernatant at their highest recommended working concentration prior to incubation with human serum IgA.

### Ig degradation assay using recombinant SpeB

Recombinant SpeB (rSpeB, FabULOUS Genovis) was activated by incubation with 5 mM DTT for 30 min at 37°C. DTT was thereafter removed twice using Zeba Spin Micro Desalting Column (Thermo Fisher Scientific) to avoid the reduction of disulfide bonds within IgA. To control complete DTT removal, a control sample was prepared by replacing rSpeB with distilled water and treated as outlined above. Activated rSpeB or a control was mixed with human serum IgA (I 4036; Sigma-Aldrich), native human IgA1 protein (ab91020; Abcam), or human native IgA2 protein (ab91021; Abcam) using 6 U rSpeB/µg of IgA and incubated for 2 hours at 37°C. To investigate the activity of rSpeB on IgA in total human serum (Biowest), the activated rSpeB (0.2, 1, and 5 U/µL) or the control was incubated in 2% human serum for 16 hours at 37°C. Samples were stored at −20°C until further processing.

### SDS-PAGE and western blot analyses

Samples for SDS-PAGE were prepared using NuPAGE^TM^ LDS sample buffer (Invitrogen), supplemented with NuPAGE Sample Reducing Agent (Invitrogen) for reducing conditions, and heated at 70°C for 10 minutes. Samples and PageRuler Plus Prestained Protein Ladder (Thermo Fisher Scientific) were loaded onto 10% or 4–12% NuPAGE Bis-Tris gels (Invitrogen) and separated by electrophoresis. Gels were stained with Imperial Protein Stain (Thermo Fisher Scientific) according to the manufacturer’s instructions or blotted to nitrocellulose or PVDF membranes (Invitrogen) using the iBlot2 dry blotting system (Invitrogen). After transfer, membranes were blocked in 5% milk in TBS with 0.05% Tween (TBS-T) for 1 hour, followed by incubation with the primary antibody in 1% milk TBS-T for 1 hour. Membranes were washed and then incubated with secondary HRP-coupled antibody for 1 hour. The following dilutions of antibodies were used: 1:5,000 anti-human IgA-HRP (A18781, Invitrogen) and 1:2,000 anti-SpeB (ab225941, Abcam). Band detection was carried out using the ChemiDoc XRS + System (Bio-Rad) or Odyssey Fc Imager (LI-COR).

### Peptide mass fingerprinting

Human serum IgA was treated with rSpeB as described above, and the degradation reaction was incubated at 37°C for 24 hours. After separation by reducing SDS-PAGE and staining, the bands of three cleavage products smaller in size than the heavy chain (HC), as well as the HC of untreated IgA, were tightly excised from the SDS-PAGE gel (SupplementaryFig. S1). Gel bands were cut into 1-mm cubes and subjected to in-gel digestion using a ProGest Investigator in-gel digestion robot (Genomic Solutions, Ann Arbor, MI, USA). Gel cubes were destained by washing with 50% acetonitrile and subjected to reduction with 5 mM DTT for 30 minutes at 56°C and alkylation with 10 mM iodoacetamide for 30 minutes before digestion with 0.002 µg of either trypsin or chymotrypsin per sample at 37°C overnight. The peptides were extracted using 5% (v/v) formic acid and concentrated to 20 µL by centrifugal evaporation (Thermo Savant SpeedVac, Thermo Fisher Scientific). Two sets of samples were either deglycosylated after the selected gel bands of interest were excised or prior to SDS-PAGE. For in-gel deglycosylation, gel pieces were destained by washing with 50% acetonitrile and then shrunk with acetonitrile before being soaked in 20 mM Tris buffer (pH 7.5) containing 50 mM NaCl and 5 mM EDTA. Rapid PNGase (New England Biolabs) was added and incubated at 50°C for 2 hours. The gel band was then treated with digestion as described below. For in-solution deglycosylation, 16 µg of human serum IgA was incubated with 2.5 µL Rapid PNGase F (New England Biolabs) following the manufacturer’s two-step protocol and then separated by reducing SDS-PAGE.

Between 1 and 10 µL of the sample (estimated from the staining intensity of the original band) was injected onto a reverse-phase PepMap trap 75 µm × 20 mm (Thermo Fisher Scientific) for pre-concentration and desalted with loading solvent (water containing 0.05% (v/v) trifluoroacetic acid) at 5 µL/min for 10 min. The peptide trap was then switched in line with the EASY-spray ES900 column (75 µm × 150 mm, 3 µm particle size). Peptides were eluted from the column using a linear solvent gradient: 4–40% of solvent B over 120 minutes, 40–60% of solvent B for 30 minutes, a sharp increase to 95% solvent B within 0.1 minutes, an isocratic hold at 95% of solvent B for 15 minutes, a sharp decrease to 2% solvent B within 0.1 minutes, and an isocratic hold at 2% solvent B for 15 minutes. Solvent A is 0.1% (v/v) formic acid in water, and solvent B is 80% (v/v) acetonitrile, 20% water, and 0.1% formic acid. The Thermo Scientific Fusion Lumos mass spectrometer was operated in DDA-positive ion mode with a cycle time of 1.5 seconds. The Orbitrap was selected as the MS1 detector, with a resolution of 120,000 and a scan range from m/z 350 to 2,000. Peptides with charge states of 2 to 7 were selected for fragmentation under DDA conditions and excluded after one occurrence for 10 seconds. Fragmentation was by HCD and ETD with ETC reaction times of 60 ms (2–3+), 40 ms (4+), and 20 ms (5–7+) for the different charge states and measured in the Orbitrap at 30,000 resolution.

The data files were searched using Mascot with combined trypsin and chymotrypsin as the cleavage enzymes and carbamidomethylation as a fixed modification of cysteines. Variable modifications of Asn to Asp (as a result of deglycosylation) and oxidation of methionine were included. Spectra were searched against an in-house database of 6,012 protein sequences to which the sequences of IgA1 sequence from structural model pdb 1iga (HC_IgA1) and IgA2 sequence from UniProt entry P0DOX2 (HC_IgA2) were added. Peptide tolerance was 20 ppm, and fragment tolerance was 0.1 Da.

### Lectin Blot

Human serum IgA was treated with rSpeB as described above, separated by reducing SDS-PAGE, and blotted to PVDF membranes. After blocking, membranes were incubated overnight at 4°C with 10 µg/mL biotinylated *Sambucus nigra* lectin (SNA; binding to sialic acid residues), 20 µg/mL biotinylated *Lens culinaris* agglutinin (LCA; binding to fucosylated tri-mannose glycans), or 5 µg/mL biotinylated *Erythrina crista-galli* lectin (ECL; binding to galactose) (all from Vector laboratories). HRP-labeled streptavidin (1:500, R&D) was used as the secondary antibody, and membranes were developed using SuperSignal West Femto Maximum Sensitivity Substrate (Thermo Fisher Scientific). Deglycosylated human serum IgA was prepared using Rapid™ PNGase F (New England Biolabs) following the manufacturer’s two-step protocol.

### Lectin ELISA

A 96-well plate was coated overnight at 4°C with 5 µg/mL IgA, IgA treated with rSpeB, or rSpeB alone in 0.05 M carbonate–bicarbonate coupling buffer (pH 9.6). Nonspecific binding sites were blocked with 1% BSA in PBS-T for 2 hours at room temperature. Afterward, the wells were incubated with 5 µg/mL lectins (see lectin blot) in 1% BSA PBS-T, followed by streptavidin–HRP (R&D Systems) for 1 hour. The plate was extensively washed with PBS-T between individual antibody incubations, and the TMB substrate reagent set (BD) was used for detection. The reaction was stopped with 1 M sulfuric acid, and absorption was measured at 450 nm and 570 nm using a microplate reader.

## RESULTS

### GAS modifies IgA in a SpeB-dependent manner

**Fig 1:**
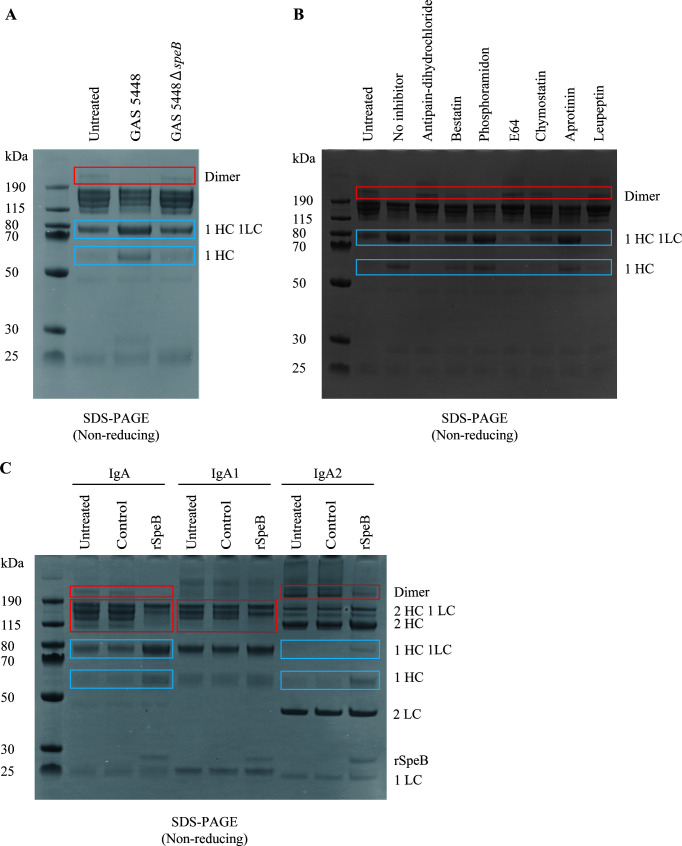
The cysteine protease SpeB modifies human serum IgA. (A) Nonreducing SDS-PAGE of human serum IgA incubated with concentrated bacterial supernatant of strain 5448 and 5448Δ*speB*. (B) Nonreducing SDS-PAGE of human serum IgA incubated with concentrated bacterial supernatant of strain 5448 in the presence or absence of indicated protease inhibitors. (C) Nonreducing SDS-PAGE of human serum IgA, IgA1, and IgA2 incubated with recombinant SpeB (rSpeB). Control indicates a sample with distilled water treated with DTT, followed by washes in an identical process as for activated rSpeB. This serves as a control to ensure complete DTT removal in the activated rSpeB. The boxed areas highlight bands that are modified by the supernatant or rSpeB. Red boxes highlight the loss of bands, while blue boxes indicate the newly appearing or intensified bands. The different forms of IgA are indicated (HC, heavy chain; LC, light chain).

To investigate the potential cleavage of human serum IgA by GAS under nonreducing conditions, we used supernatants prepared from overnight cultures of GAS strain 5448, a well-characterized invasive *emm*1 isolate (17), and its isogenic mutant strain 5448Δ*speB* (18). Western blot analyses of the bacterial supernatant confirmed that GAS 5448 expressed SpeB, including the mature proteolytic form (Fig. S2). SDS-PAGE analysis of IgA treated with concentrated supernatant of 5448 showed a loss of higher molecular weight forms of IgA, including the dimeric form, while intensified bands with a mass of approximately 75 kDa likely corresponding to that of heavy chain (HC) and light chain (LC) and a newly appearing band agreeing with the expected 55 kDa mass of HC monomers, as compared to untreated IgA (Fig. 1A). In contrast, concentrated bacterial supernatants of 5448Δ*speB* failed to modify IgA. As SpeB is a cysteine protease, a panel of protease inhibitors was tested. This showed that the four cysteine protease inhibitors (antipain dihydrochloride, E64, chymostatin, and leupeptin) completely abolished IgA cleavage, while no effect was noted for the other protease inhibitors (Fig. 1B), further supporting a SpeB-dependent effect.

To further evaluate the IgA modification by SpeB, recombinant SpeB (rSpeB) was incubated with human serum IgA, purified IgA1, or purified IgA2. Human serum IgA showed a similar but more pronounced cleavage following rSpeB treatment, compared to that observed with 5448 supernatants (Fig. 1C). Different cleavage patterns were seen for IgA1 and IgA2 (Fig. 1C). In IgA1, the multimeric (2HC) band was lost, while no other modifications could be observed. In contrast, in IgA2, the 2HC band remained intact, whereas the dimeric form was lost, and intensified bands at 55 and 75 kDa were noted. To reduce the effect of interchain oligomerization affecting the band pattern, reducing SDS-PAGE gel separation was used to further evaluate rSpeB proteolytic cleavage of IgA. Three protein bands of lower apparent mass than HC could be observed (Fig. S1). The modification products of IgA were only slightly smaller in size than the HC (∼55–60 kDa), suggesting that SpeB does not target the hinge region. In addition, the activity of rSpeB in total human serum showed a concentration-dependent IgA cleavage (Fig. S3).

### SpeB cleavage results in loss of C-terminal regions of IgA

**Fig 2:**
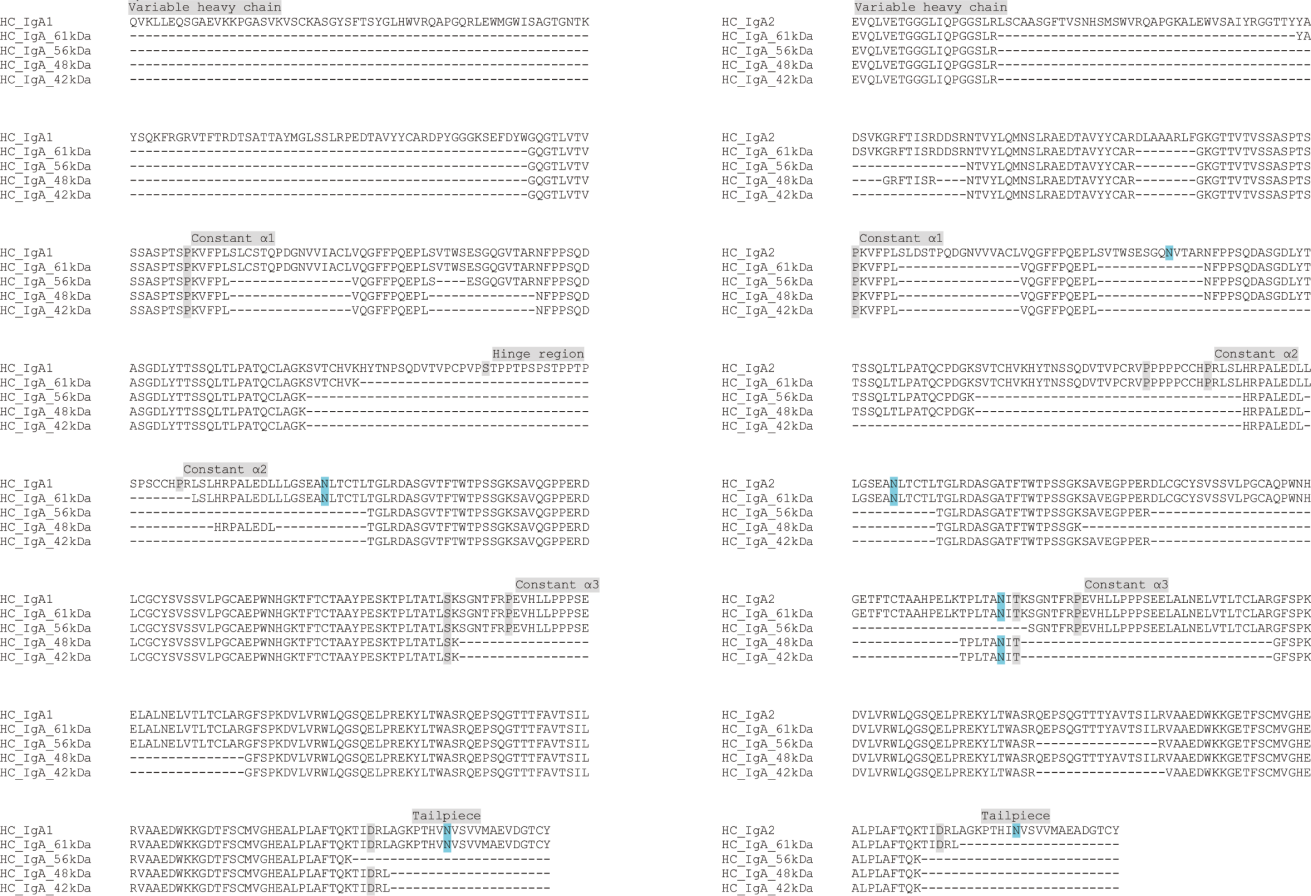
Terminal modification of human serum IgA by rSpeB. Multiple amino acid sequence alignments of peptide fingerprint hits generated by mass spectrometry of untreated IgA heavy chain (HC_IgA_61 kDa) and modified IgA products (HC_IgA_56 kDa, HC_IgA_48 kDa, and HC_IgA_42 kDa). IgA1 sequence from structural model pdb 1iga (HC_IgA1) and IgA2 sequence from UniProt entry P0DOX2 (HC_IgA2) were used as references. Domain boundaries are highlighted in grey and N-glycosylation sites in cyan.

The degradation of higher molecular weight forms of IgA suggests a preference for the cleavage of multimeric serum IgA and an impact on dimerization. The modification products were smaller in size than HC, suggesting a modification different from the classical hinge cleavage. To gain insight into the SpeB cleavage site in IgA, mass spectrometry (MS) analysis was performed on the excised gel bands of the three smaller modification products (indicated by asterisks, (Fig. S1). To maximize the coverage of IgA sequences in peptide mass fingerprinting, we used in-gel digestion with trypsin and chymotrypsin. Coverage was further extended through in-gel and in-solution digestion of samples with the deglycosylase PNGase F. The analysis was limited by the scarce sequence information for the variable HC domains (19) and the polyclonal nature of serum IgA. Sequences specific for both IgA1 and IgA2 were detected in all gel bands analyzed (Fig. 2). No coverage for the variable HC domain of IgA1 was obtained. However, a unique N-terminal peptide matching IgA2 was observed in all analyzed bands of in-solution PNGase F/trypsin digests, suggesting that all observed fragments resulted from rSpeB-mediated cleavage at the C-terminal region. The C-terminal tailpiece region contains one of the N-glycosylation sites and was expected to be detectable only in peptide mass fingerprinting of deglycosylated samples. However, it was only covered in one of our searches, the control HC band in an in-solution PNGase F/trypsin digest. This suggests that this region did not readily give rise to a peptide amenable to MS or that deglycosylation with PNGase F was ineffective. Comparative analyses of overlapping amino acid sequences from the untreated HC and all the modification products suggest a putative cleavage at the C-terminal region, including the tailpiece and a N-glycosylation site. Although unlikely, an N-terminal modification of IgA1 by rSpeB cannot be excluded.

### SpeB modification affects the glycosylation of IgA

**Fig 3:**
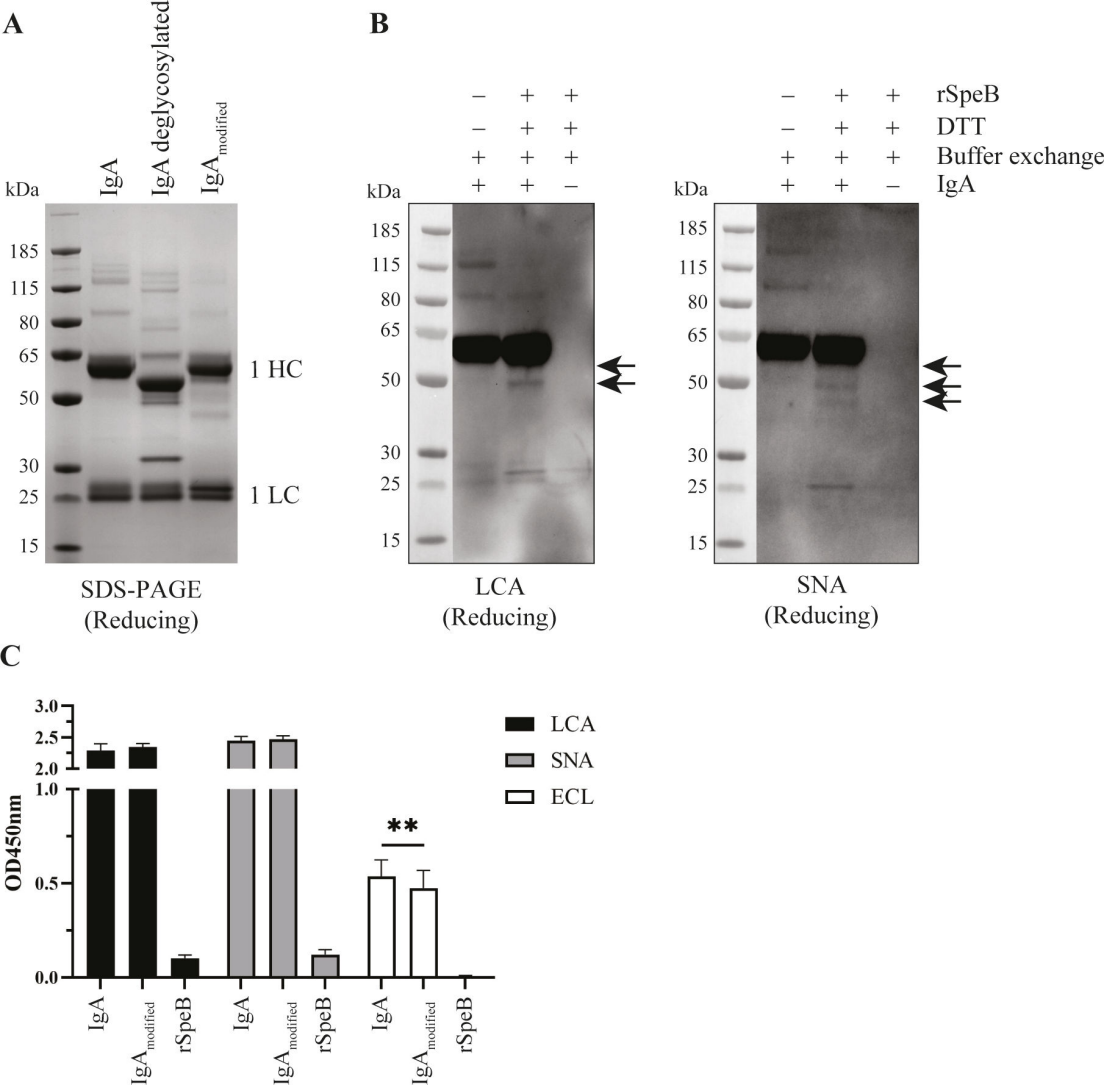
IgA modification by rSpeB influences the glycosylation profile. (A) Reducing SDS-PAGE of deglycosylated human serum IgA. An additional band of 36 kDa in deglycosylated IgA corresponds to PNGase F. HC, heavy chain; LC, light chain. (B) Lectin blot of human serum IgA modified by recombinant SpeB (rSpeB) using *Lens culinaris* agglutinin (LCA) and *Sambuccus nigra* lectin (SNA). The addition of DTT is required for initial rSpeB activation. Buffer exchange indicates the removal of DTT before incubation with IgA. The protein ladder was loaded on the same gel as the samples, transferred to a PVDF membrane, and imaged using the 700 nm channel. (C) Lectin ELISA of untreated or modified human serum IgA incubated with lectins LCA, SNA, and *Erythrina crista-galli* lectin (ECL) (*n* = 3). Significance was tested with a paired *t*-test. **P < 0.01.

The modification implicated at the conserved C-terminus of IgA HC likely results in the loss of the tailpiece, which is known to be involved in dimer formation (20). In addition, the tailpiece possesses one N-glycosylation site per HC (N340 in IgA1 and N327 in IgA2) (21). To explore this, the glycosylation profile of human serum IgA modified by rSpeB was investigated (Fig. 3). Comparison of the SDS-PAGE band patterns of deglycosylated IgA and untreated IgA showed the expected shift in molecular weight corresponding to the loss of N-glycans (Fig. 3A). However, IgA modified by rSpeB shows a different migration pattern than deglycosylated IgA, consistent with the modification not constituting a complete removal of attached glycans.

The binding of lectins LCA and SNA, which are specific for core glycans and terminal sialic acid, respectively, was analyzed by lectin blot. This indicated the loss of higher molecular weight glycosylated species and the presence of remaining N-glycosylation in SpeB-modified IgA (Fig. 3B). Notably, the smallest 42 kDa IgA modification product was detected only with sialic acid-binding SNA, but not with the fucosylated tri-mannose binding LCA. ECL, which specifically binds to galactose, showed no signal in the lectin blot (data not shown), indicating a sensitivity issue. To increase detection sensitivity, the binding of lectins to IgA was additionally investigated by lectin ELISA (Fig. 3C). In the ELISA, all three lectins showed binding to IgA, and consistent with the Western blot results, LCA and SNA showed higher OD values than ECL. There was no difference in the binding of LCA or SNA to untreated or rSpeB-modified IgA. However, ECL binding was slightly decreased in modified IgA, suggesting less galactose in these samples. Although the decrease was small, it was highly consistent and in agreement with only a portion of the IgA being modified by SpeB.

## DISCUSSION

Specialized IgA1 proteases have been reported for many pathogens, including several *Streptococcus* species, such as *S. pneumoniae*, *S. oralis*, and *S. mitis*, but not for GAS despite its ability to efficiently colonize and infect mucosal surfaces. In the present study, we demonstrate that human serum IgA is modified by GAS in a SpeB-dependent manner. However, only a minor portion of the serum IgA was affected, and the cleavage pattern and the mass spectrometry analysis indicate that SpeB targets the C-terminus rather than the hinge region. The data further indicates that SpeB predominantly affects IgA2.

The function of SpeB as an Ig-degrading enzyme has been debated. SpeB was initially reported as a specific IgG hinge region cleaving enzyme, which could also cleave the HC of IgA, IgM, IgD, and IgE (15). Moreover, a recent study suggested two additional cleavage sites at the Cα2–Cα3 interface of IgG (22). Since the activation of the cysteine protease depends on reducing conditions (23), the Ig degradation assays used in the report by Collin and Olsén (15) were performed under such conditions during which Ig cleavage was observed. However, in a subsequent study, the Ig-degrading activity of SpeB was explored under nonreducing conditions (16). In this study, no SpeB cleavage of Ig could be noted. This showed the importance of removing the reducing agent used for SpeB activation through buffer exchange to allow a more physiological conformation of IgA without the reduction of disulfide bridges.

In our study, which was also performed under nonreducing conditions, the modification products of human serum IgA by SpeB were smaller in size than the HC and differed from the classical hinge cleavage products. Our MS data imply that the modification affects the C-terminal tailpiece. This is in line with the previously reported sequential C-terminal cleavage of IgA, which leads to the loss of the tailpiece as well as the Cα3 and Cα2 HC domains by *Proteus mirabilis* proteinase (24, 25). Also, a serine proteinase from *Staphylococcus aureus* cleaves secretory IgA1 and myeloma IgA2 at multiple sites distinct from the hinge region (26). Our attempts to determine the SpeB cleavage site in IgA by MS analysis were hampered by the incomplete sequence coverage of the untreated control IgA HC. The observed differences in the apparent size of SpeB-modified IgA fragments, compared to the untreated IgA, ranged from 5 to 19 kDa. The loss of N-glycosylation sites can strongly influence the migration pattern of polypeptides. Thus, minor modifications can result in greater differences in electrophoretic mobility on SDS-PAGE. In theory, partial or complete loss of one terminal domain of the IgA HC could be possible. However, the cleavage profile with an intensified 75 kDa band and a newly emerging 55 kDa band, combined with the loss of the high molecular weight band likely representing dimeric IgA, is identical to the banding pattern shown for IgA mutants with tailpiece deletion (27). This is also in agreement with the tailpiece being demonstrated to have a critical role in the IgA dimerization (20). Also, the glycosylation profiles indicated slightly, but significantly, less galactose in the IgA sample modified by SpeB compared to untreated IgA. The observed difference is likely mediated by the proteolytic modification by SpeB rather than by a classical deglycosylase activity.

In summary, our results demonstrate that the streptococcal cysteine protease SpeB modifies human serum IgA, resulting in the loss of multimeric and dimeric forms of IgA—an effect seen predominantly in IgA2. Although studies have suggested a protective role of IgA in GAS colonization and infection through impaired bacterial adherence and neutralization of toxins (28, 29), there are, to our knowledge, no reports on IgA1 and IgA2 in streptococcal humoral responses. As dimeric IgA2 is the predominant form in human mucosa, more pro-inflammatory than IgA1 (21), and critical in the neutralization of pathogens and toxins at this site, it is tempting to speculate that IgA modification by SpeB represents an immune evasion strategy utilized by GAS to colonize human mucosal tissue. However, this remains to be elucidated in future studies to address the functional consequences of SpeB cleavage of IgA, particularly IgA2, with respect to GAS colonization and infection.

## SUPPLEMENTAL MATERIAL

Supplemental figures spectrum.02450-24-s0001.pdf Figures S1 to S3.

ASM does not own the copyrights to Supplemental Material that may be linked to, or accessed through, an article. The authors have granted ASM a non-exclusive, world-wide license to publish the Supplemental Material files. Please contact the corresponding author directly for reuse.
