# Supplementary material for: Group A streptococcal SpeB modifies IgA through targeting regions other than the hinge
Source: Microbiol Spectr. 2025 Mar 25;13(5):e02450-24. doi: 10.1128/spectrum.02450-24 (PMC12054102; doi:10.1128/spectrum.02450-24)
Supplement: Supplemental figures — Figures S1 to S3. [file spectrum.02450-24-s0001.pdf]

Supplemental material

Title: Group A streptococcal SpeB modifies IgA through targeting regions other than the hinge

Authors: Victoria Vassen, Emi Tanaka, Kirsten Moll, Christian Spoerry, Silvia Synowsky, Sally L Shirran, Ulrich Schwarz-Linek, Edmund Loh, Mattias Svensson, Anna Norrby-Teglund

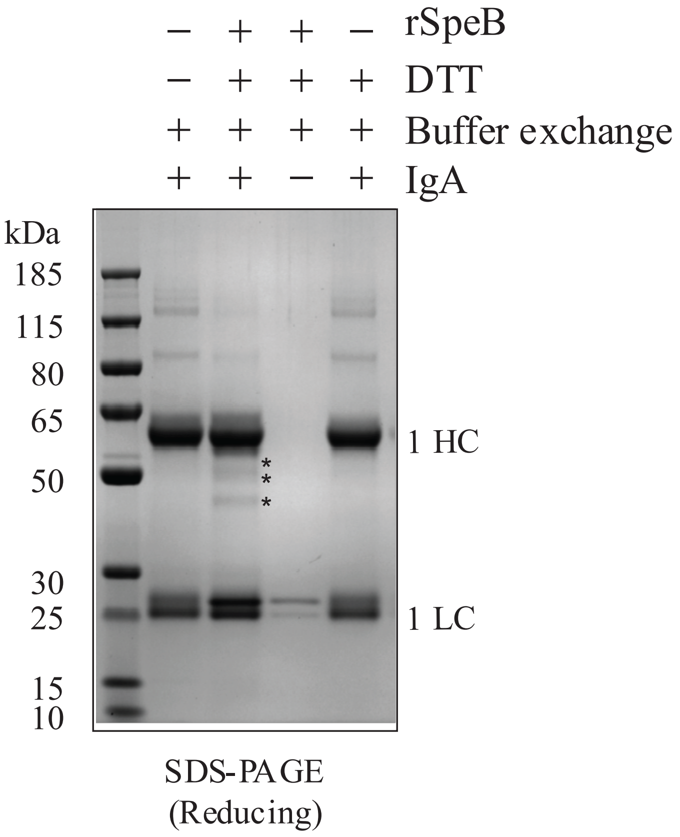

Supplemental Figure 1. Human serum IgA modification by rSpeB assessed by reducing SDS-PAGE.

Reducing SDS-PAGE of human serum IgA incubated with recombinant SpeB (rSpeB). Addition of DTT is required for initial SpeB activation. Buffer exchange indicates the removal of DTT before incubation with IgA. Asterisks indicate IgA modification products (approx. 56 kDa, 48 kDa and 42 kDa). HC, heavy chain; LC, light chain.

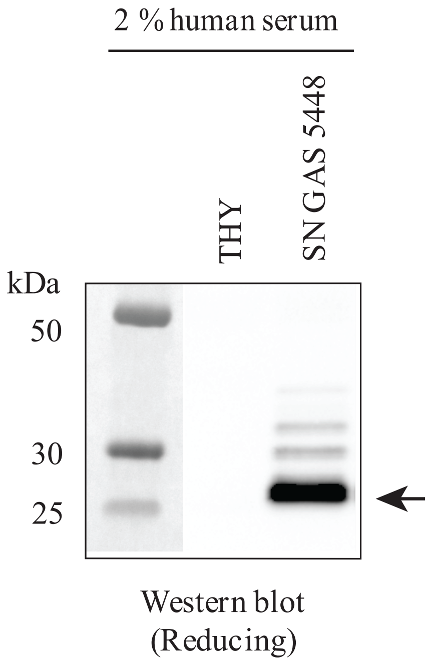

Supplemental Figure 2. SpeB expression in GAS 5448 supernatants (SN).

Western blot analysis of bacterial culture SN collected after growth in THY medium in 2 % human serum. The blots were exposed to anti-SpeB antibodies. The arrow highlights the mature form of SpeB. The protein ladder was loaded on the same gel as the samples, transferred to a PVDF membrane, and imaged using the 700 nm channel.

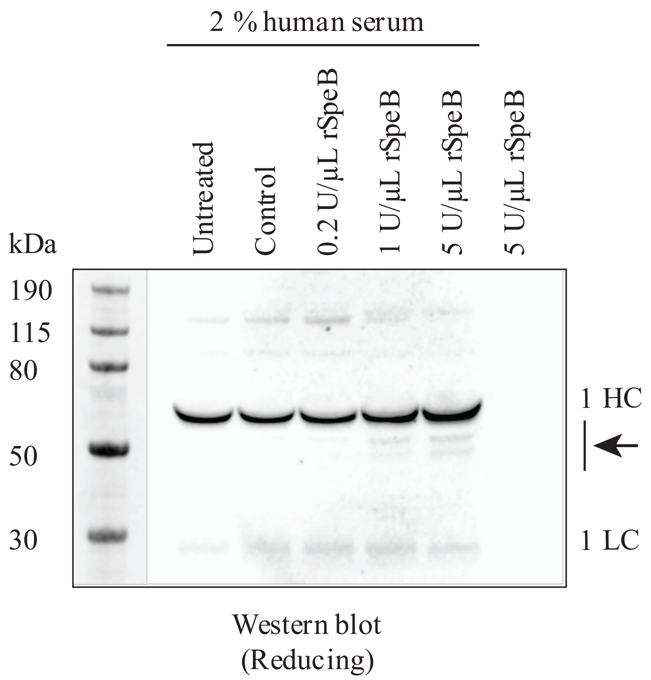

Supplemental Figure 3. SpeB modifies IgA in total human serum.

Western blot analysis of 2 % human serum mixed with rSpeB. The blots were exposed to anti-human IgA antibodies. Control indicates a sample prepared with distilled water replacing rSpeB to ensure complete DTT removal in the activated rSpeB. The arrow highlighted bands that are modified by rSpeB. The protein ladder was loaded on the same gel as the samples, transferred to a nitrocellulose membrane, and imaged using the 700 nm channel. HC, heavy chain; LC, light chain.
